# Impact of Likelihood Ratios of Rheumatoid Factor and Anti-Cyclic Citrullinated Peptide Antibody in Clinical Diagnosis of Rheumatoid Arthritis by Two Available Platforms

**DOI:** 10.3390/diagnostics15020135

**Published:** 2025-01-08

**Authors:** Juan Irure-Ventura, María Díaz-Toledo, Noelia Palazuelos-Cayón, Marcos López-Hoyos

**Affiliations:** 1 Immunology Department, University Hospital Marqués de Valdecilla, 39008 Santander, Spain; juan.irure@scsalud.es; 2 Immunopathology Group, University Hospital Marqués de Valdecilla-IDIVAL, 39011 Santander, Spain; 3 Faculty of Medicine, University of Cantabria, 39011 Santander, Spain; maria.diaz-toledo@alumnos.unican.es (M.D.-T.); noelia.palazuelos@alumnos.unican.es (N.P.-C.)

**Keywords:** rheumatoid arthritis, rheumatoid factor, anti-cyclic citrullinated peptide antibodies, likelihood ratio

## Abstract

**Background/Objectives**: Rheumatoid arthritis (RA) is one of the most prevalent autoimmune diseases, characterized by an articular and extra-articular involvement, where autoantibodies, such as rheumatoid factor (RF) and anti-cyclic citrullinated peptide antibodies (ACPAs), are important biomarkers for the diagnosis. Autoantibody determination can be carried out using different assays. However, the results obtained are usually expressed in arbitrary units that are not comparable. Therefore, the aim of this study is to improve clinical interpretation of RF and ACPA test results using the likelihood ratio (LR). **Methods**: RF and ACPA titers were analyzed by turbidimetry and chemiluminescence using Optilite and BIO-FLASH systems, respectively, in 781 samples from patients with RA and in 1970 controls. **Results**: The higher the antibody titer of RF or ACPA, the higher the LR for RA. The definition of test result interval-specific LR based on predefined specificities for antibody levels provides more information than the use of the cut-off set by the manufacturer for each antibody. **Conclusions**: The LR for RA increased with an increasing antibody level. In addition, the use of test result interval-specific LR allows better clinical interpretation for RF and ACPA assays compared to the traditional idea of interpreting antibody results in a dichotomous manner, such as negative or positive.

## 1. Introduction

Rheumatoid arthritis (RA) is one of the most common chronic inflammatory diseases. It mainly affects the joints, although it must be considered a syndrome that includes extra-articular manifestations such as rheumatoid nodules, pulmonary involvement, or vasculitis, as well as systemic comorbidities [1,2,3].

The incidence of RA ranges from 0.5% to 1%, with an apparent decrease from north to south countries and a decrease in urban regions compared to rural ones [1].

RA is pathologically heterogeneous, existing as an environmental exposure or triggering factor acting in genetically predisposed patients. Most genetic associations are observed in individuals with the disease and positive levels of ACPA [4]. In fact, the production of ACPA is associated with HLA-DRB1, whose expression is a risk factor for the production of these antibodies, but not an independent risk factor for the development of the disease [5].

ACPAs are a prominent feature of RA, and their presence increases the risk of RA compared to RF [6,7]. Its positivity can be used to classify patients into two subgroups, namely,

- Seropositive RA (with the presence of ACPA and/or IgM RF);
- Seronegative RA (with absence of ACPA and IgM RF).

These constitute two different disease patterns given the underlying pathophysiology [2,3,8,9].

Different phases in the development of RA are currently recognized, the last of which is the moment when the diagnosis of RA is established. ACPA can be identified years before clinical symptoms manifest, in the preclinical phases [3]. Over the course of the disease, their concentration and diversity increase in the same way as their serum levels.

According to the current 2010 European Alliance of Associations for Rheumatology (EULAR) and American College of Rheumatology (ACR) international classification standards for RA, for an early diagnosis of RA, the patient’s initial evaluation should be based on a detailed history and physical examination. This evaluation should, in turn, support the suspicion of the disease with the determination of RF, ACPA, acute phase reactants, and imaging techniques [10].

The measurements of ACPA and RF, on which the diagnosis of the disease is based, are included in the current criteria due to their specificity and high prevalence in patients who suffer from it [10,11]. However, although serology contributes to increasing the sensitivity and specificity of these standards in the early diagnosis of the disease, the use of these criteria is not recommended in all patients [12].

Therefore, depending on whether or not arthritis is seropositive,

- In patients with seropositive RA, it is recommended, with a recommendation level of grade B, to use the ACR/EULAR 2010 classification criteria as a support for the initial clinical impression.
- In contrast, in patients with seronegative arthritis, a medical opinion will be more useful than the application of the new classification standards.

Considering the great importance given to serological status in the 2010 ACR/EULAR classification criteria for RA, the relevance of seropositivity against ACPA specifically is not surprising, since seronegative patients take longer to receive a diagnosis and also have greater disease activity [8]. Despite this, there is still a significant level of variation between the methods used to standardize the levels of RF and ACPA, which has an impact on the diagnostic capacity of these criteria. In a meta-analysis in which the diagnostic accuracy of RF and ACPA was analyzed, the sensitivity of RF and ACPA for RA diagnosis was similar, 69% and 67%, respectively. However, the presence of ACPA showed a higher specificity (95%) than RF (85%) [13].

To ensure consistency across the variety of available techniques, cut-off values should be aligned by defining them based on a predefined specificity in a control cohort. Higher levels of RF and ACPA have been shown to be found more frequently in RA patients. It should be noted that when physicians order a laboratory test without a clear diagnosis in mind, a test result should help with the differential diagnosis. However, the measured quantity will not directly indicate which possible diagnoses will have higher probability. Nevertheless, the associated disease-specific likelihood ratio (LR) for each of the possible diseases will allow for weighing them. In addition, the use of LR would help the harmonization of diagnostic tests, specifically in autoimmunity, where quantitative results are expressed in arbitrary units that are not comparable [14,15]. In this sense, the establishment of LR in the determination of RF and ACPA is a good strategy to normalize the results obtained, since there is not a good agreement among the currently available assays [16,17,18]. The work carried out by Van Hoovels et al. has been essential in establishing the importance of LR for RF and ACPA in RA patients, taking into account especially the comparison performed between different assays [19].

The aim of this study is to improve the clinical interpretation of RF and ACPA test results using the LR based on Optilite and BIO-FLASH results, two platforms that have not been evaluated to date for this purpose, and to establish its application in the prediction and diagnosis of RA.

## 2. Materials and Methods

### 2.1. Study Population

A retrospective observational study was carried out including one serum sample obtained between 1 January 2018 and 31 December 2022 from 2751 patients, in which RF and ACPA levels were determined at the Immunology Service of the Marqués de Valdecilla University Hospital. This research was conducted in accordance with the Declaration of Helsinki and was approved by the Regional Ethics Committee (CEIm Internal code: 2023.43). The clinical record of each patient was reviewed to determine if the patient presented RA or not, in order to establish the RA cohort (n = 781) and control cohort (n = 1970). The control cohort was made up of individuals who presented osteoarthritis (n = 124), psoriatic arthritis (n = 58), and spondyloarthritis (n = 48), as well as patients with interstitial lung disease (n = 58), systemic lupus erythematosus (n = 38), Sjögren’s syndrome (n = 20), antiphospholipid syndrome (n = 16), or other systemic autoimmune rheumatic diseases (n = 142), and healthy subjects (n = 1446). The specific data of the subjects included in RA and control cohorts considering the presence of RF and/or ACPA are depicted in Table 1.

### 2.2. Quantification of RF and ACPA

All samples were analyzed using the same methodology following the manufacturer’s protocols.

RF quantification was performed by immunoturbidimetry using an Optilite RF IgM kit (The Binding Site, Birmingham, UK), whereas ACPAs (anti-CCP3 antibodies) were determined by chemiluminescence using a QUANTA Flash CCP3 kit on a BIO-FLASH system (Werfen, San Diego, CA, USA).

### 2.3. Statistical Analysis

The diagnostic yield of the methodologies used for the determination of RF and ACPA (anti-CCP3 antibodies) was evaluated by calculating the sensitivity, specificity, and likelihood ratio (LR) for each defined interval.

In order to define the intervals in a consistent manner in the different methodologies, they were delimited by thresholds corresponding to predefined specificities (90%, 95%, 97.5%, 99%, and 99.9%), as well as the one obtained using the cut-off recommended by the manufacturer in each case.

Statistical analyses were carried out using MEDCALC (V.17.1, Ostend, Belgium) and Graph Pad Prism 6 software.

## 3. Results

### 3.1. Analytical Performance of RF Assay According to Manufacturer and Laboratory Cut-Offs

Considering the cut-offs established by the manufacturer and the laboratory, Table 2 shows the characteristics of the analytical performance of the RF assay on our study population.

Taking into account the cut-off established by the manufacturer, the system has a fairly high sensitivity, which is achieved by implementing a low cut-off point. In order to improve the specificity of the assay, the laboratory considered it necessary to increase the cut-off point, preserving a relatively good sensitivity.

Considering the ACR/EULAR 2010 classification criteria for RA, a patient will have high positivity for RF when the values are three times higher than the reference value or threshold of the laboratory and/or assay, which would correspond in this case, with levels higher than 37.5 or 66 IU/mL when the cut-offs determined by the manufacturer of the laboratory are applied, respectively. The analytical performance characteristics of the RF assay applying three times higher manufacturer and laboratory cut-offs are shown in Table 3.

Considering these new cut-offs, a significantly higher specificity is achieved with both strategies, reaching levels higher than 90%, and therefore, a higher positive LR for RA is obtained.

### 3.2. Test Result Interval-Specific LR for RF Assay

Once we verified the behavior of the assay using the cut-off points defined by the manufacturer and the laboratory, we analyzed how the LR for RA is modified depending on the levels of RF. In order to determine test result intervals in a consistent manner, in addition to those corresponding to the cut-offs established by the manufacturer and the laboratory, we defined different intervals delimited by thresholds that correspond to predefined specificities (90.0%, 95.0%, 97.5%, 99.0%, and 99.9) for RF.

**Figure 1:**
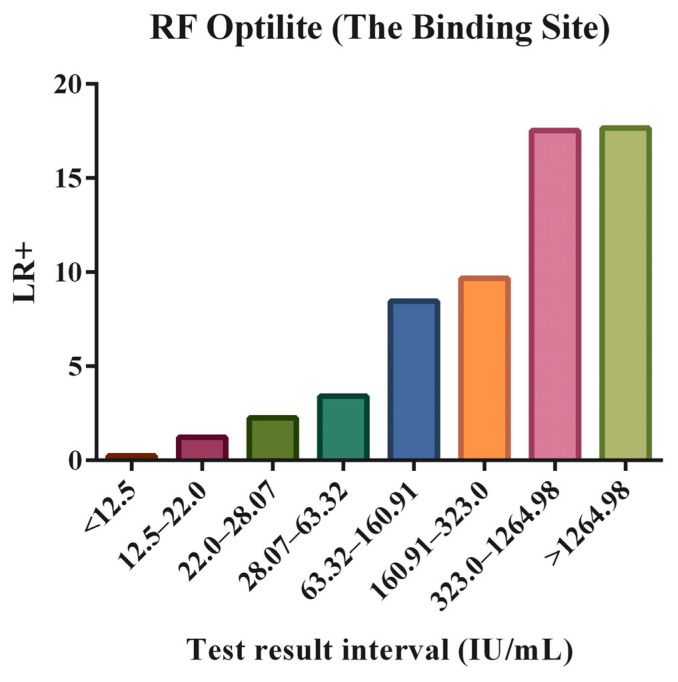
The positive likelihood ratio (LR+) of the rheumatoid factor (RF) for the different test result-specific intervals, delimited by thresholds that correspond to predefined specificities (90.0%, 95.0%, 97.5%, 99.0%, and 99.9), in addition to those corresponding to the cut-offs established by the manufacturer and the laboratory.

In each of these defined intervals, the positive LR for RA with the 95% CI was calculated, as well as the number of patients with RA and controls that presented RF levels within them. The obtained results are depicted in Table 4 and illustrated in Figure 1.

As is expected, Figure 1 clearly shows that the higher the antibody titer, the higher the LR for RA. The last two intervals seem to show very similar positive LRs for RA, which is due to the fact that in the last interval, the number of patients included is limited.

### 3.3. Analytical Performance of ACPA Assay According to Manufacturer and Laboratory Cut-Offs

Unlike what we observed in the case of RF, in the quantification of ACPA, the laboratory uses the cut-off point recommended by the manufacturer. Considering this aspect, the analytical performance characteristics for ACPA determination are shown in Table 5.

As in the case of RF, the cut-off point defined by the manufacturer to establish ACPA positivity is relatively low and as a consequence, the sensitivity obtained is high but the specificity is moderate.

Defining a new cut-off point at three times that established by the manufacturer (60 IU/mL) in order to identify patients with high ACPA titers according to ACR/EULAR 2010 classification criteria for RA, we observed that with this new cut-off point, the specificity of the assay increases considerably, exceeding 90%, and consequently the LR+ is also notably high, which suggests that patients with high levels of ACPA are approximately nine times more likely to have RA than those without high levels (Table 6).

### 3.4. Test Result Interval-Specific LR for ACPA Assay

Once we analyzed the behavior of the assay using the cut-off points defined by the manufacturer and applied by the laboratory, we analyzed how the LR for RA is modified depending on ACPA titers.

Again, in order to determine test result intervals in a consistent manner, in addition to those corresponding to the cut-offs established by the manufacturer and the laboratory, we defined different intervals delimited by thresholds that correspond to predefined specificities (90.0%, 95.0%, 97.5%, 99.0%, and 99.9) for ACPA.

**Figure 2:**
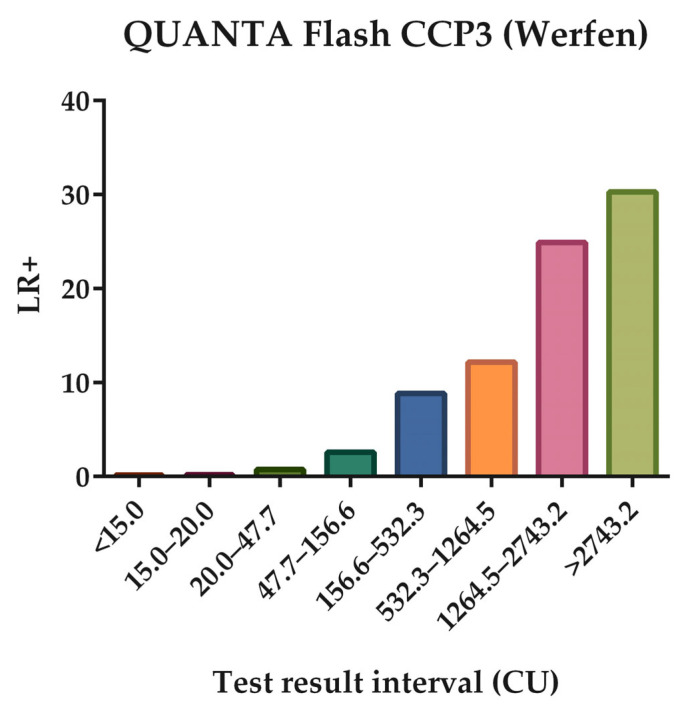
The positive likelihood ratio (LR+) of anti-citrullinated peptide antibodies (ACPAs) for the different test result-specific intervals, delimited by thresholds that correspond to predefined specificities (90.0%, 95.0%, 97.5%, 99.0%, and 99.9), in addition to the cut-off established by the manufacturer and applied by the laboratory.

In each of these defined intervals, the positive LR for RA with the 95% CI was calculated, as well as the number of patients with RA and controls that presented RF levels within them. The obtained results are depicted in Table 7 and illustrated in Figure 2.

Despite the cut-off established by the manufacturer, allowing us to identify patients at risk of developing RA, as it is shown in Table 7 and Figure 2, the higher the ACPA titers, the higher the positive LR for RA, and specifically LR increases significantly for an interval greater than 90% of specificity.

## 4. Discussion

The use of LR defined as the fraction of patients with a particular test result to the fraction of controls with the same test result [14,20,21] is a good strategy to harmonize the interpretation of autoantibody results using commercial assays for their determination including those for RF and ACPA, since the concept of LR is a method that is independent of the units used to express the results [14,21]. In this sense, a test result with an LR of 10 indicates that this test result is 10 times more likely to be found in patients with the disease than in controls, whereas a test result with an LR of 0.1 is 10 times less likely to be found in patients with the disease than in controls. Therefore, the LR allows us to convey immediately clinically relevant information related to the antibody level. The higher the antibody level, the higher the LR for disease [16,22].

Nowadays, RF and ACPA are the two autoantibodies included in the 2010 ACR/EULAR classification criteria for RA [10], but despite the fact that they have been used for many years, there are still doubts regarding the interpretation of the results obtained, since there is a great amount of tests available for their determination using different technologies [16].

In recent years, the use of LR has extended to the interpretation of different autoantibodies, such as anti-nuclear antibodies (ANAs) [23], anti-cytoplasmic neutrophil antibodies (ANCAs) [24], anti-tissue transglutaminase [25], RF, or ACPA [19,22,26], among others, and begins to be incorporated into clinical practice.

Focusing on improving the clinical interpretation of RF and ACPA results by the use of LR, a recently published paper showed that using the cut-offs proposed by the manufacturer, there was a large variability in diagnostic sensitivity and specificity between assays for RF and ACPA determination. However, the implementation of test result interval-specific LRs was concordant across the different RF and ACPA assays that were tested, showing that for all assays, the LR for RA increased with an increasing antibody level. Therefore, the definition of these thresholds for antibody levels and assigning test result interval-specific LRs allowed the alignment of clinical interpretation for the different RF and ACPA assays [19].

Based on the results obtained in this study, we defined the LR according to the RF and ACPA results obtained using two platforms that to date had not been tested for this purpose, including Optilite and BIO-FLASH, respectively, and we observed that the results are comparable and therefore, we could align interpretation across companies, and thus overcome the limitation that arises as a consequence of the use of different units and cut-off points. The differences in the LR observed between the two systems that were evaluated and the ones published [19] could be due to characteristics of the patients included in the control cohort.

In this sense, the traditional idea of interpreting the result of an autoantibody in a dichotomous way, as negative or positive, must be put aside and clinicians should begin to think about different probabilities of experiencing an autoimmune disease depending on the levels of autoantibodies through the use of test result-specific LRs, which contain more clinically relevant information.

As it has been shown, the higher the antibody level of RF or ACPA, the higher the LR for RA, and this LR is independent of the assay used. It is important that laboratory professionals and clinicians become more familiar with the concept of LRs and that they develop an intuitive feeling for the clinical relevance of an LR [20].

## 5. Conclusions

The LR for RA increased with an increasing antibody level. In addition, the use of test result interval-specific LR allows better clinical interpretation for RF and ACPA assays compared to the traditional idea of interpreting antibody results in a dichotomous manner, such as negative or positive. The use of LR should be extended among laboratory specialists, and clinicians should be aware of its value for the better interpretation of autoantibody results in patients with autoimmune diseases.

## Figures and Tables

**Table 1:** Subjects included in RA and control cohorts considering the presence of RF and ACPA.

Cohort	Total n (%)	RF− ACPA− n (%)	RF+ ACPA− n (%)	RF− ACPA+ n (%)	RF+ ACPA+ n (%)
RA	781 (28.4)	63 (8.1)	29 (3.7)	136 (17.4)	553 (70.8)
Control	1970 (71.6)	1382 (70.2)	122 (6.2)	353 (17.9)	113 (5.7)
HS	1446 (73.4)	1048 (72.5)	85 (5.9)	245 (16.9)	68 (4.7)
OA	124 (6.3)	81 (65.3)	4 (3.2)	24 (19.4)	15 (12.1)
PsA	78 (4.0)	54 (69.2)	4 (5.1)	15 (19.2)	5 (6.4)
ILD	58 (2.9)	32 (55.2)	6 (10.3)	12 (20.7)	8 (13.8)
SpA	48 (2.4)	33 (68.8)	3 (6.3)	10 (20.8)	2 (4.2)
SLE	38 (1.9)	17 (44.7)	6 (15.8)	11 (28.9)	4 (10.5)
SS	20 (1.1)	10 (50.0)	7 (35.0)	2 (10.0)	1 (5.0)
APS	16 (0.8)	9 (56.3)	2 (12.5)	4 (25.0)	1 (6.3)
Other SARD	142 (7.2)	98 (69.0)	5 (3.5)	30 (31.1)	9 (6.3)

Abbreviations—RA: rheumatoid arthritis; RF: rheumatoid factor; ACPA: anti-cyclic citrullinated peptide antibody; HS: healthy subject; OA: osteoarthritis; PsA: psoriatic arthritis; ILD: interstitial lung disease; SpA: spondyloarthritis; SLE: systemic lupus erythematosus; SS: Sjögren’s syndrome; APS: antiphospholipid syndrome; SARD: systemic autoimmune rheumatic disease. % reflects the percentage of subjects in each subgroup. The cut-offs used to consider positivity for RF and ACPA were 22 IU/mL and 20 CU, respectively.

**Table 2:** Performance characteristics of the RF assay at the cut-offs defined by the manufacturer and the laboratory.

	RF Optilite (The Binding Site) Manufacturer Cut-Off	RF Optilite (The Binding Site) Laboratory Cut-Off
Units	IU/mL	
Measuring range	7–6500	
Cut-off	12.5	22
Sensitivity (%)	80.41 (77.4–83.1)	74.52 (71.3–77.5)
Specificity (%)	83.3 (81.6–84.9)	88.07 (86.6–89.5)
LR+	4.81 (4.34–5.35)	6.25 (5.50–7.09)
LR−	0.24 (0.20–0.27)	0.29 (0.26–0.33)

Abbreviations—RF: rheumatoid factor; LR: likelihood ratio; IU: international units. The 95% confidence intervals are presented within brackets.

**Table 3:** Performance characteristics of RF assay applying three times higher manufacturer and laboratory cut-offs.

	RF Optilite (The Binding Site) Manufacturer Cut-Off	RF Optilite (The Binding Site) Laboratory Cut-Off
Units	IU/mL	
3 × Cut-off	37.5	66
Sensitivity (%)	65.43 (62.0–68.8)	51.98 (48.4–55.5)
Specificity (%)	91.73 (90.4–92.9)	95.23 (94.2–96.1)
LR+	7.91 (6.77–9.24)	10.89 (8.84–13.42)
LR−	0.38 (0.34–0.42)	0.50 (0.47–0.54)

Abbreviations—RF: rheumatoid factor; LR: likelihood ratio; IU: international units. The 95% confidence intervals are presented within brackets.

**Table 4:** Test result-specific LR for RF.

	Interval	n (%), RA	n (%), Control	LR+	95% CI, LR+
RF Optilite (The Binding Site) CO = 22 IU/mL	<12.5	153 (19.59)	1641 (83.30)	0.235	0.204–0.271
	12.5–22.0	46 (5.89)	95 (4.82)	1.221	0.867–1.720
	22.0–28.07	33 (4.23)	37 (1.88)	2.250	1.417–3.571
	28.07–63.32	134 (17.16)	99 (5.03)	3.414	2.669–4.367
	63.32–160.91	161 (20.61)	48 (2.44)	8.461	6.196–11.553
	160.91–323.0	115 (14.72)	30 (1.52)	9.669	6.526–14.327
	323.0–1264.98	125 (16.01)	18 (0.91)	17.517	10.762–28.511
	>1264.98	14 (1.79)	2 (0.10)	17.657	4.022–77.511

Abbreviations—RF: rheumatoid factor; CO: cut-off; IU: international units; RA: rheumatoid arthritis; LR: likelihood ratio; CI: confidence interval.

**Table 5:** Performance characteristics of the ACPA assay at the cut-off defined by the manufacturer.

	QUANTA Flash CCP3 (Werfen)
Units	CU
Measuring range	4.6–2776.7
Manufacturer cut-off	20
Sensitivity (%)	88.22 (85.7–90.4)
Specificity (%)	76.35 (74.4–78.2)
LR+	3.73 (3.43–4.05)
LR−	0.15 (0.13–0.19)

Abbreviations—ACPA: anti-citrullinated protein antibody; CCP: cyclic citrullinated peptide; LR: likelihood ratio; CU: chemiluminescence units. The 95% confidence intervals are presented within brackets.

**Table 6:** Performance characteristics of ACPA assay applying three times higher manufacturer cut-off.

	QUANTA Flash CCP3 (Werfen)
Units	CU
Manufacturer cut-off	60
Sensitivity (%)	75.42 (72.2–78.4)
Specificity (%)	91.62 (90.3–92.8)
LR+	9.00 (7.74–10.48)
LR−	0.27 (0.24–0.30)

Abbreviations—ACPA: anti-citrullinated protein antibody; CCP: cyclic citrullinated peptide; LR: likelihood ratio; CU: chemiluminescence units. The 95% confidence intervals are presented within brackets.

**Table 7:** Test result-specific LR for ACPA.

	Interval	n (%), RA	n (%), Control	LR+	95% CI, LR+
QUANTA Flash CCP3 (Werfen) CO = 20 CU	<15.0	80 (10.24)	1333 (67.66)	0.151	0.123–0.187
	15.0–20.0	12 (1.54)	173 (8.78)	0.175	0.098–0.312
	20.0–47.7	78 (9.99)	267 (13.55)	0.737	0.581–0.935
	47.7–156.6	102 (13.06)	99 (5.03)	2.599	1.996–3.383
	156.6–532.3	172 (22.02)	49 (2.49)	8.854	6.518–12.028
	532.3–1264.5	145 (18.57)	30 (1.52)	12.192	8.302–17.904
	1264.5–2743.2	168 (21.51)	17 (0.86)	24.927	15.242–40.767
	>2743.2	24 (3.07)	2 (0.10)	30.269	71.171–127.77

Abbreviations—ACPA: anti-citrullinated protein antibody; CCP: cyclic citrullinated peptide; RA: rheumatoid arthritis; LR: likelihood ratio; CI: confidence interval; CO: cut-off; CU: chemiluminescence units.

## Data Availability

The raw data supporting the conclusions of this article will be made available by the authors on request.
